# Neurology of cancer immunotherapy

**DOI:** 10.1007/s10072-022-06297-0

**Published:** 2022-09-16

**Authors:** Amedeo De Grado, Federica Cencini, Alberto Priori

**Affiliations:** Clinical Neurology Unit, San Paolo University Hospital, Department of Health Sciences and “Aldo Ravelli” Research Center for Experimental Brain Theraputics, University of Milan, ASST Santi Paolo e Carlo, Milan, Italy

**Keywords:** Neurological complications, NAEs, Cancer immunotherapy, Meningoencephalitis, Myelitis, Demyelinating disorders

## Abstract

**Background:**

Immunotherapy is nowadays considered a mainstay of cancer treatment, dramatically affecting the disease-free survival rate in several aggressive malignancies. Unfortunately, cancer immunotherapy can also trigger life-threatening autoimmune neurological complications named “neurological adverse effects” (*NAEs*). *NAEs* can affect both the central nervous system (CNS), as in ipilimumab-related aseptic meningitis, and the peripheral nervous system (PNS), as in nivolumab-induced myasthenia gravis.

**Current evidence:**

The incidence of *NAEs* is highly variable, ranging from 2 to 4% using checkpoint inhibitors to 50% using blinatumomab. Looking at these numbers, it appears clear that neurologists will soon be called more and more frequently to decide upon the best therapeutic strategy for a patient receiving immunotherapy and experiencing a *NAE.* Most of them can be treated or reverted withholding the offending drug and adding IVIg, plasmapheresis, or steroids to the therapy. Sometimes, however, for oncological reasons, immunotherapy cannot be stopped so the neurologist needs to know what countermeasures have proven most effective. Moreover, patients with a pre-existing autoimmune neurological disease (*AID*), such as myasthenia gravis or multiple sclerosis, might need immunotherapy during their life, risking a severe worsening of their symptoms. In that setting, the neurologist needs to properly counsel patients about the risk of a therapy-related relapse.

**Conclusion:**

In this article, we describe the most frequently reported *NAEs* and aim to give neurologists a practical overview on how to deal with them.

## Introduction

Immunotherapy has revolutionized the treatment of several types of cancer and is nowadays considered a first-line treatment in many different scenarios. As a matter of fact, cancer immunotherapy has led to favorable outcomes in terms of tumor regression and patient survival in several malignancies. The tumors that have benefited the most from immunotherapy are non-small cell lung cancers (with better overall survival, response rates, and disease-free survival) [1], cutaneous melanoma (in which anti–CTLA-4 monoclonal antibodies increased the 10-year survival rate from 10 to 22%) [2] and hematological cancers [3]. Immunotherapy works by activating the immune system, and, by doing so, inducing an anti-tumor response [4]. The most frequently used immunotherapeutic agents act as follows:

1. Immune checkpoint inhibitors (ICIs): monoclonal antibodies that target the cytotoxic T-lymphocyte antigen-4 (CTLA-4) or programmed cell death-1/receptor-1 (PD-1/PD-L1) cancer cell pathways, allowing T cells to remain activated and attack malignant cells [5]
2. Chimeric antigen receptor (CAR)-T cell therapy: patient’s T cells equipped with recombinant proteins that allow them to recognize targets on cancer cells, independently from the MHC complex [6]
3. Therapeutic cancer vaccines: exogenous administration of selected tumor antigens, generally combined with adjuvants, in order to stimulate the patient’s adaptive immune system against specific tumor antigens. Different approaches are the use of autologous tumor lysates, whole tumor-derived mRNA, irradiated autologous tumor cells, or allogeneic tumor cell lines [7]
4. Bispecific T-cell-engaging (BiTE) antibodies: antibody constructs with two binding domains that link endogenous T cells to tumor-expressed antigens, thus activating the cytotoxic potential of a patient’s own T cells against malignant cells [8]

Even if immunotherapy is considered safer than conventional therapy (chemotherapy and targeted therapy), like any therapeutic entity, it is not risk free. It can be responsible of a unique range of immune-related adverse events (*irAEs*) in terms of organ involvement, onset pattern, and severity [9]*.* The incidence of *irAEs* varies among different drugs, doses, time of exposure, and types of malignancies [10]. Whereas most *irAEs* are generally mild, some authors have reported severe and sometimes even life-threatening *irAEs* which led to therapy discontinuation. *IrAEs* affecting the nervous system (*NAEs*) are underrecognized but well-known entities with a multifaceted range of presentation, and since the use of immunotherapeutic agents will increase over time, neurologists shall become familiar with this ever-expanding new chapter of neurology.

By more accurately characterizing the neurotoxicity, physicians will be able to better guide the work-up, treatment, and management of neurological complaints.

In this review, we provide an overview on the commonest immunotherapy-associated *NAEs*; as for ICIs, we will mainly focus on nivolumab-, pembrolizumab-, and ipilimumab-related *NAEs* due to their wide use and the vast experience matured during these years, but *NAEs* such as myositis have been described also using newer ICIs (i.e., tislelizumab) [11]. Most of therapeutic cancer vaccines are currently being investigated in clinical trials, and only few of them have been approved by the FDA; hence, definite data about their adverse effects profile are missing.

We will further discuss current evidence about pathogenesis, epidemiology, core clinical characteristics, and prognosis of most common *NAEs*. Finally, we conclude with fragments of management recommendations.

## Mechanisms of immunotherapy neurotoxicity

The underlying pathogenesis of *NAEs* remains largely unclear, but several possible mechanisms implicated in neurotoxicity have been advocated. As for ICIs, some authors formulated a “tumor-induced epitope” theory consisting in a cross-reactivity between antigens expressed on tumoral cell (or released by the tumor after cell destruction) and normal self-antigens expressed on neural tissues. The autoimmune response seems to be mainly cell-mediated (via T cell and macrophage activation), causing damage to virtually all components of the nervous system. Other factors contributing to the pathogenesis include genetic predisposition, pre-existing autoimmune diseases, and environmental factors [12]. For example, several studies emphasize the possibility for ICIs-related encephalitis to be mainly anti-Ma2 antibody mediated and, less frequently, anti-NMDAR and anti-Hu mediated, similarly to what happens in paraneoplastic syndromes [13, 14]. Other, less characterized, mechanisms of *NAEs* will be briefly discussed within the text. Systemic toxicity induced by CAR-T cell therapy is related to tumor lysis syndrome (TLS) and cytokine release syndrome (CRS), both caused by a disorganized immunologic response towards neoplastic cell destruction induced by the therapy [15]. Neurotoxicity could be an indirect effect related to cytokines entrance into the brain by passive diffusion from the circulation, or it could be a consequence of cytokines produced by intrathecal CAR-T cells [16].

**Fig. 1:**
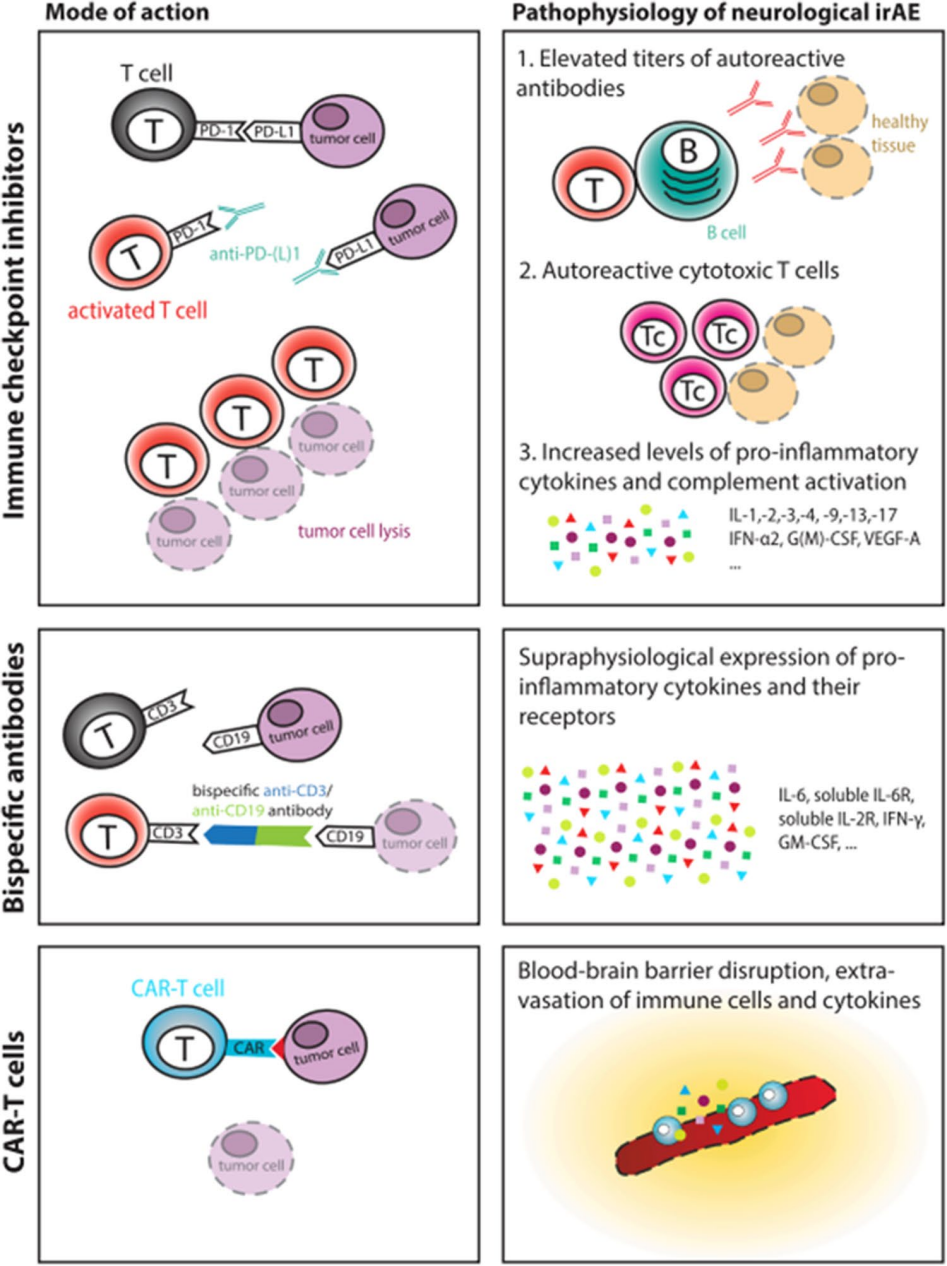
Pathophysiology of immune-related neurological complications. The presumed mechanisms leading to neurological complications in the context of treatment with ICI, bispecific antibodies, and CAR T cells are shown. Abbreviations: IL, interleukin; IFN, interferon; GM-CSF, granulocyte–macrophage colony-stimulating factor; VEGF, vascular endothelial growth factor (with permission from Roth P. et al. “Neurological complications of cancer immunotherapy.” [18])

The BiTE-associated neurotoxicity is thought to be related to activated T cells that adhere to the endothelium, cross the blood–brain barrier (BBB), and enter the CNS with subsequent B cells activation and cytokine release, eventually leading to BBB damage and symptoms’ onset [17, 18]. Current knowledge about the *NAEs*’ pathophysiology is summarized in Fig. 1.

### Adverse neurological reactions to immunotherapy

It is now widely accepted that *NAEs* can involve both the CNS and the PNS. The overall incidence of *NAEs* using ICIs is highly variable, ranging from 0.2 to 14% in patients treated with pembrolizumab/nivolumab and from 0.3 to 0.8% in those receiving ipilimumab [19], with the highest rates associated with combination of nivolumab plus ipilimumab [20, 21]. More specifically, grade 1 and 2 adverse effects such as headache, dizziness, and paresthesia manifest in 6–12% of patients, rarely determining the discontinuation of therapy [22], whereas grade 3 and 4 *NAEs*, such as inflammatory myopathies, myasthenia gravis, aseptic meningitis, autoimmune encephalitis, multiple sclerosis, and posterior reversible encephalopathy syndrome (PRES) manifest in fewer than 1% of cases, often requiring therapy withdrawal. According to Larkin et al., the combination of anti-CTLA4 and anti-PD-1 drugs caused a detrimental effect in a percentage ranging between 2.4 and 14% of treated patients [23]. From a pharmacovigilance study, analyzing data from 18,518,994 patients who experienced *NAEs* after ICI therapy emerged that encephalomyelitis and meningitis affect younger patients compared to myasthenia gravis and Guillain-Barré syndrome (58.6 and 56.3 versus 70.3 and 65 years old). The malignancies most commonly associated with the onset of *NAEs* were lung cancer (33% of all cases; *n* = 188/574) and melanoma (36%; *n* = 206/574) [24].

In a 2014 published study including 36 patients treated with blinatumomab (a BiTE), the occurrence of a *NAE* resulted in treatment discontinuation in 17% of cases. Of the six patients who experienced a *NAE*, five were classified as having a grade 3 adverse event consisting of tremor, aphasia, confusion, seizures, or a combination thereof [25]. Other studies have shown that therapy with blinatumomab causes an overall neurological toxicity (central and peripheral) in 60% of treated patients, with an average onset time of 2 weeks. Common blinatumomab-induced *NAEs* were tremor (resting, intentional, and essential), lethargy, mental status changes (i.e., stupor and sleepiness), dizziness, seizures (mostly clonic and atonic), aphasia, hypoesthesia, trigeminal neuralgia, abducens, and facial nerve paralysis [26].

### *Centralnervous system involvement*

#### Encephalitis

- **ICIs:** The overall incidence seems to be around 0.84% [27]. From a clinical review conducted in 2017 emerged that 6 out of 3763 patients treated with ICI experienced encephalitis. All of them received nivolumab (± ipilimumab) for advanced melanoma; 1 of these 6 events was fatal despite aggressive corticosteroid therapy. Of the remaining 5 cases, 4 resolved without sequelae in 5–21 days after therapy discontinuation, whereas the fifth only marginally improved after drug suspension [23]. In another study including 48,653 patients, encephalitis and/or myelitis were reported in 250 patients (0.5%) [24]. The average age at onset varies between 58.6 and 65 years, and the mean latency between onset and death has been reported to be 60.8 days [13, 24]. In a case series of 9 patients receiving either pembrolizumab, nivolumab, or atezolizumab, the most frequently reported symptoms were confusion (78%), fever (45%), and cerebellar ataxia (33%); none of them had seizures [28].
- **Blinatumomab**: Its tolerability has been evaluated in a study including 189 patients treated for B-cell acute lymphoblastic leukemia in adults with minimal residual disease. The mean onset time was 2 weeks [26]. Overall, encephalopathy was reported in 10 patients (5%), aphasia in 7 (4%), mental status change in 7 (4%), seizures in 4 (2%), and hemiparesis in 2 (1%). Unfortunately, no specifics were given on the term “encephalopathy,” and no further tests were performed [25, 26]. Insights on the pathogenetic mechanism are lacking. The exact pathogenetic mechanism is yet to be elucidated, but it is probably due to blinatumomab-activated T cells crossing the BBB and binding to CD19 + B cells which in turn release cytokines and disrupt the BBB [29].
- **CAR-T cell therapy**: Several *NAEs* have been observed in patients treated with CAR-T cell therapy, which can be combined under the name *CRES* (CAR-T cell-related encephalopathy syndrome) and include delirium, hallucinations, altered consciousness, tremors, ataxia, aphasia, myoclonus, drowsiness, and seizures. They can also cause cerebral edema, a disorder that in a few patients has proven fatal [30]. In a long-term follow-up of 53 patients with relapsed B-cell ALL, grade 2 neurotoxic effects arose in 2% of cases, grade 3 in 36%, and grade 4 in 6%, with a greater risk in patients with high disease burden (> 5% blasts in bone marrow or extramedullary disease) [31]. The time at onset can vary from 1 day up to 3–4 weeks after therapeutic infusion. Toxicity is generally reversible and, in most cases, resolves spontaneously, although it may sometimes require immunosuppressive therapy and intensive care. Although the pathogenesis remains unclear, the severity of neurotoxicity correlates with that of CRS, the most common adverse effect. However, the two events do not necessarily develop simultaneously, suggesting an independent pathogenesis but with shared causative factors [30].

#### Aseptic meningitis

- **ICIs:** ICI-related aseptic meningitis seems to arise typically within the first 7 weeks of treatment and in 0.1–0.2% of patients [23, 32]. Aseptic meningitis was most frequently observed in patients started on anti-CTLA-4 rather than anti-PD-1/PD-L1 agents and peculiarly for metastatic cutaneous melanoma rather than metastatic lung cancer. The average time-to-onset was 3 months, while the time-to-death (when occurred) was 42 days. The mean age at onset was 56.3 years [24].

#### Multiple sclerosis (MS) and demyelinating diseases

- **ICIs:** Considering the immune-mediated genesis of MS, it is not surprising that it may be exacerbated by ICIs therapy [33, 34]. In one case, ipilimumab was found responsible for transition from radiologically isolated syndrome (RIS) to clinically definite MS. This patient received two courses of ipilimumab 2 years apart, and after each administration, he experienced either the onset of neurological deficits or the appearance of new lesions on MRI. After treatment, his MS remained stable with IFN-b treatment [35]. The literature shows that up to 30% of patients with MS who receive treatment with ICI experience worsening of their symptoms or a new, generally mild, immunological event [34]. Garcia et al. identified 14 patients with a previous history of MS who had a relapse while receiving ICIs; the median age was 52.5 years, and the median time-to-onset of symptoms was 29 days, with rapid disease progression. The median time for symptom resolution was 8 weeks. Nivolumab was the most associated with MS (*n* = 9), followed by ipilimumab (*n* = 5), pembrolizumab (*n* = 2), and atezolizumab (*n* = 1). No cases were attributed to durvalumab or avelumab [34]. Other demyelinating pathologies such as optic neuritis and transverse myelitis can manifest from weeks to months after treatment with pembrolizumab, nivolumab, and ipilimumab [36]. The causal relationship is poorly understood, but one of the proposed mechanism states that PDL-1 is expressed on astrocytes and microglia surface during inflammatory conditions and that ICIs administration, by preventing the physiological switch-off mediated by the PD-1/PDL-1 complex, leads to an increase in inflammatory response within the CNS [36].

#### Posterior reversible encephalopathy syndrome (PRES)

**Fig. 2:**
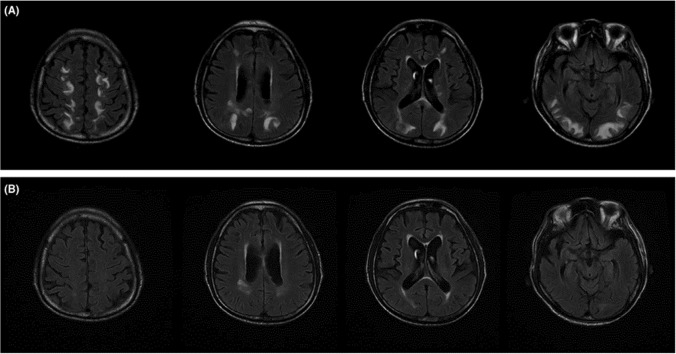
ICIs-related posterior reversible encephalopathy syndrome. **A** T2 FLAIR (fluid‐attenuated inversion recovery)‐weighted magnetic resonance imaging showing subcortical and deep white matter high signal intensity involving both cerebral hemispheres, especially temporooccipital and high frontoparietal areas. **B** Two‐week follow‐up study showing decreased extent of high signal intensity lesions in subcortical and deep white matter of both cerebral hemispheres (with permission from Kim D. et al. “Posterior reversible encephalopathy syndrome induced by nivolumab immunotherapy for non–small-cell lung cancer”. [38])

- **ICIs:** Mostly nivolumab and pembrolizumab, less frequently ipilimumab. Combined therapy may increase the likelihood of PRES [37–39]. To date no systematic literature reviews have addressed the cumulative incidence of this side effect, being probably less than 1% of treated patients (Fig. 2).

#### Transverse myelitis

- **ICIs:** Liao et al. described a 62-year-old patient with a history of uveal melanoma who developed lower extremity weakness, Babinski sign, sensory level at T10 as well as intermittent urinary retention and fecal incontinence during treatment with ipilimumab. Thoracic spine MRI without contrast showed a focal T2 signal abnormality at T9-10 without swelling of the cord. All the myelopathy work-up was negative; hence, ipilimumab-related transverse myelitis was suspected, and the drug was withheld [40]. Nivolumab has recently proven to be responsible for transverse myelitis aswell [41], while pembrolizumab has been associated with a longitudinal extensive transverse myelitis resembling NMOSD on imaging [42].

### Peripheral nervous system involvement

#### Myasthenia gravis (MG)

- **ICIs**: A 2017 study analyzed a cohort of 10,277 patients with malignancy treated with either ipilimumab or nivolumab and found 12 cases of de novo MG. All cases arose during treatment with nivolumab (nivolumab-induced MG or nivo-MG), while no de novo MG was diagnosed during ipilimumab treatment. No apparent gender preference (6 men and 6 women) emerged from the analysis, and the mean onset time was between 7 and 11 weeks. Ten out of 12 nivo-MG patients had anti-acetylcholine receptor antibodies, and none had anti-MuSK. Moreover, 105 patients with idiopathic myasthenia gravis (iMG) were selected as controls, and data were compared between the two groups (nivo-MG vs iMG)*.* The analysis showed a higher prevalence of bulbar symptoms and myasthenic crises and an overall more severe disease course assessed with the MGFA classification in patients with nivo-MG rather than iMG. Patients in the nivo-MG group also had markedly elevated creatine kinase (CK) levels, with a mean of 4.799 IU/L, suggestive of a concomitant immune-mediated polymyositis, and 3 of them were diagnosed with myocarditis due to the abrupt onset of heart failure or arrhythmias (with proven lymphocyte infiltration on cardiac muscle biopsy) [43]. A pharmacovigilance study published in 2019 highlighted other features of ICI-induced myasthenia gravis: first, it confirmed the PD-1 tendency to induce MG (197 cases) compared to CTLA-4 (14 cases) or combined therapy (17 cases); second, it reported an average ICI-induced onset time of 29 days, considerably shorter than previously thought and shorter than any other neurological side effect observed in the same study (Guillain-Barré, aseptic meningitis, and encephalitis); and third, it described MG as having the most fulminant presentation, with a nearly 20% fatality rate and a frequent association with myositis and myocarditis (16 and 10%, respectively) [24]. The suggested mechanism for MG development after ICIs administration is epitope-sharing between the tumor and the healthy tissue which leads to antibody production and cytotoxic T-cell activation toward common epitopes [44, 45].

#### Peripheral neuropathies

- **ICIs:** The incidence of peripheral neuropathies is extremely variable. Overall, ICIs induced neuropathy in less than 5% of the registered cases. Data suggest that ICI-associated neuropathy is considerably higher among patients receiving the combination of CTLA-4 and PD-1 inhibitors (1.6%) compared to patients receiving PD-1 inhibitors alone (0.3%) [46]. Among these neuropathies, we recognize small fiber neuropathy and cranial neuropathies as well as more complex immune-mediated syndromes such as Guillain-Barré-like syndrome (GBS-like, 0.1–0.25% of cases) or chronic inflammatory demyelinating polyneuropathy (CIDP) [40, 46, 47]. In a series of 19 patients receiving ipilimumab, nivolumab, pembrolizumab, and atezolizumab (either alone or in combination) and who developed neuropathies, cranial neuropathies were the most commonly reported (*n* = 7, mostly involving the facial nerve and bilateral in 1 case), followed by non-length-dependent polyradiculoneuropathies (*n* = 6), small-fiber/autonomic neuropathy (*n* = 2), sensory neuronopathy (*n* = 1), neuralgic amyotrophy (*n* = 1), ANCA-associated mononeuritis-multiplex (*n* = 1), and length-dependent sensorimotor axonal polyneuropathy (*n* = 1) [46]. Despite aggressive treatment, two cases of GBS-like syndrome were fatal. Symptoms commonly arise about a week after drug administration. The presence of mitigating factors explaining an acute onset in some patients and a chronic one in others is still a matter of debate [19, 47]*.* Clinically, patients present with a wide variety of symptoms ranging from acute/chronic polyradiculo-neuropathies to meningo-radiculo-neurites, cranial mononeuropathies (optic, abducens, or facial diplegia) or multi-neuropathies [19, 23, 40]. However, the incidence of peripheral neuropathy of any degree using ICIs seems to be significantly lower than with conventional chemotherapy [44].
- **Blinatumomab:** The overall incidence of neuropathy using blinatumomab is extremely low, and it is not clearly correlated with the cumulative administered dose. In a 2018 study conducted enrolling 189 patients, 7 experienced paresthesia, 6 experienced hypoesthesia, 3 had dysesthesia, 1 had facial nerve palsy, and, mostly notably, 39 patients developed tremor. Unfortunately, no specifics were given on the nature of these disturbs, and no further tests were performed in most cases [48].

#### Myositis and myocarditis

- **ICIs:** In one of the largest reported cohorts, the median onset of symptoms was 25 days after therapy’s first course (5–87 days) [49]. Weakness involves mostly neck flexors and proximal girdle muscles, and patients only rarely require invasive ventilation due to respiratory muscles involvement [50]. Of note, oculomotor and bulbar muscles may be affected in ICIs-related myositis, mimicking MG, hence, accurate differential diagnosis is warranted to properly treat the patient [50]. As for myocarditis, in 2018, Al-Kindi and colleagues identified 250 cases of ICI-related myocarditis, half of which resulted in death (124 patients), making myocarditis the most fearsome of all ICI-related *irAEs*. The overall incidence of myocarditis seems to be low (0.76%), but the fatality rate is concerning. Many authors did not find any any differences between PD/PDL-1 and CTLA-4, but combined or sequential therapy may lead to an increased risk [51].

## Immunotherapy in patients with pre-existing neurological autoimmune diseases

The use of immunotherapy in patients with pre-existing autoimmune diseases (*AIDs*) is highly controversial. Experience is limited since they have been regularly excluded from clinical studies presuming that they would have been at greater risk of developing severe *NAEs*.

Abdel-Wahab et al. conducted a systematic review on this topic and gathered data on 123 patients with ICIs-treated cancer and concomitant *AIDs*. The review reported a broad range of pre-existing *AIDs*, including multiple sclerosis and myasthenia gravis. About 33% of patients with multiple sclerosis had exacerbation of symptoms, and they improved or remained stable after treatment with corticosteroids. All patients with myasthenia gravis had an adverse event, three of them had exacerbation of the disease, and the other one experienced an adverse event unrelated to his pre-existing *AID*. Adverse events improved with corticosteroids, immunoglobulin, plasmapheresis, anticholinesterase inhibitors, and/or rituximab.

Patients receiving any therapy for their *AIDs* before starting ICIs seemed to have fewer adverse events than those not receiving any treatment (59% vs. 83%) [52]*.*

A retrospective analysis reported a patient with metastatic melanoma and an underlying NMOSD who developed a longitudinal extensive transverse myelitis with severe clinical symptoms after two doses of ipilimumab. Corticosteroids did not improve the clinical outcome [53]*.*

In conclusion, though pre-existing *AIDs* are not an absolute contraindication for ICIs therapy, these agents may lead to their exacerbations. Clinicians should carefully assess the individual risk of each patient considering the type, severity, and activity of pre-existing *AIDs* and consider alternative treatment options, when available.

## Diagnosis

Given the wide clinical spectrum of *NAEs*, it is not possible to draw up a unique protocol for diagnosis, therapy, and prognosis even though the diagnostic work-up for most of *NAEs* differs little from their non-immunotherapy-related counterparts. Brahmer and colleagues provided a useful diagnostic algorithm for ICIs-related *NAEs* which could be applied also to non-ICI *NAEs*, at least until more specific guidelines will be approved (Table 1). Interestingly, when dealing with immunotherapy-related MG, it is always advisable to perform an electrocardiogram as a first-line screening to rule out a possible concomitant myocarditis, even in asymptomatic patients. As for ICI-related myositis, classic myositis-specific antibodies do not represent a reliable tool to confirm or exclude the diagnosis as they are often negative, but muscle biopsies demonstrating endomysial infiltration of CD68 + cells expressing PD-L1 and CD8 + lymphocytes expressing PD-1 as well as sarcolemmal upregulation of MHC-1 may help orienting the diagnosis [54].

**Table 1:** A practical overview on the most common NAEs. ^1^Mild (grade 1) neurologic symptoms do not generally require immunotherapeutic agent withdrawal and may be continued under close observation. For grade 2 (or higher) *NAEs*, drug suspension is recommended in addition to pharmacologic therapy. Acronyms: *AChR* acetylcholine receptor. *CK* creatine kinase, *CSF* cerebrospinal fluid, *ENMG* electroneurography/electromyography, *GBS* Guillain–Barre syndrome, *HIV* human immunodeficiency virus, *ICIs* immune checkpoint inhibitors, *IVIg* intravenous immunoglobulin, *MRI* magnetic resonance, *MuSK* muscle-specific tyrosine kinase, *PEX* plasma exchange, *TSH* thyroid stimulating hormone

	**Incidence**	**Time to onset**	**Clinical features**	**Differential diagnosis**	**Diagnostic work-up**	**Treatment**^**1**^	**Prognosis**
Encephalitis	-ICIs: 0.84% -Blinatumomab: 5% -CAR-T cell: grade 2: 2%, grade 3: 36% grade 4: 6%	CAR-T cell: 1–28 days	- ICIs: confusion, fever, cerebellar ataxia -CAR-T cell: hallucinations, altered consciousness, tremors, ataxia, aphasia, myoclonus, drowsiness and seizures	Infection; metabolic disturbances; brain metastasis; leptomeningeal dissemination; cerebrovascular infarct; intracerebral hemorrhage	-MRI head ± spine with contrast -CSF analysis -Antibodies research (Ma-2, NMDAR, anti-Hu, CASPR2, LGI1, MOG AQP4 or others as suggested by the clinical picture)	ICIs suspension + high dose steroids ± IVIg or plasma exchange (PEX) Blinatumomab: Tocilizumab (anti IL-6R)	Generally improves spontaneously after treatment suspension
Meningitis	ICIs: 0.1–0.2%	49 days	Headache, neck stiffness, nausea, photophobia, emesis, delirium and altered consciousness, coma	Infection; metabolic disturbances; leptomeningeal dissemination	-MRI head ± spine with contrast -CSF ﻿analysis	High-dose steroids (rule out bacterial or viral infections prior to high dose steroids)	Generally responsive to therapy
Multiple sclerosis	30% of patients with previous MS	29 days	Relapse: worsening of symptoms or a new immunological event	Infection, metabolic disturbances	-MRI head ± spine with contrast	500 mg/1 g intravenous methylprednisolone for 3–5 days	14 patients: -5 symptoms resolution -3 MS progression -2 death
PRES	ICIs: less than 1%	17 days from the last dose (10–24 days)	Headache, confusion, visual changes, seizures and focal neurological deficits associated with high blood pressure levels	Infection; metabolic disturbances; brain metastasis; leptomeningeal dissemination; cerebrovascular infarct; intracerebral hemorrhage	-MRI head ± spine with contrast -CSF ﻿analysis -Antibodies ﻿research (Ma-2, NMDAR, anti-Hu, CASPR2, LGI1, MOG, AQP4 or others as suggested by the clinical picture)	ICIs suspension + high dose steroids ± IVIg or PEX	Generally responsive to therapy
Transverse myelitis	Case reports for ICIs	-	Sensory/motor/bowel and bladder changes below a certain spinal cord level	Spinal metastasis; spinal cord compression	-MRI head ±spine with contrast -CSF ﻿analysis -Blood test (B12, HIV, RPR, ANA, Ro/La, TSH, aquaporin-4 immunoglobulin G) -Antibodies (Ma-2, CASPR2, anti-Hu, LGI1, MOG, AQP4 or others as suggested by the clinical picture)	ICIs suspension + high dose steroids IVIg or PEX if steroid unresponsive	Generally responsive to therapy
Neuropathies	ICIs: less than 5% Blinatumomab: case reports	GBS: 7 days	ICIs: cranial neuropathies, small fiber neuropathy, polyradiculoneuropathies, sensory neuronopathy, neuralgic amyotrophy, ANCA-associated mononeuritis-multiplex and polyneuropathies	Metabolic; toxic (previous chemotherapy, vitamin deficits); leptomeningeal dissemination	-ENMG -Serum: exclude diabetes, B12, folate deficiency, HIV, TSH, consider vasculitis and autoimmune screen -Consider CSF analysis -Consider MRI of the lumbar spine/roots	-Pregabalin, gabapentin and duloxetine as symptomatic drugs -GBS: drug suspension plus steroid. PEX or IVIg if steroid unresponsive -Polyradiculoneuropathy or meningo-radiculoneuritis: corticosteroids at high doses or IVIg	Generally responsive to therapy
Myasthenic syndromes	ICIs: mostly nivolumab	29 days (49–77 days)	Ptosis, ophthalmoparesis, diplopia, dysphagia, dysarthria, facial muscle weakness, neck, and/or respiratory muscle weakness	Inherited and metabolic myopathies; toxin-induced myasthenic syndrome (D-penicillamine, chloroquine); polymyositis; Lambert-Eaton syndrome	-Serum: Anti AChR and anti-MUSK antibodies -Repetitive nerve stimulation and single-fiber EMG -Spirometry -ECG	-High-dose steroids -Pyridostigmine -If unresponsive: plasmapheresis, IVIG, azathioprine, cyclosporine, and mycophenolate may be considered	Fatality rate: Isolated MG (16.2%); isolated myositis (20.7%); isolated myocarditis (33%) MG + Myocarditis and myositis (62.5%)
Myositis	0.76%	5–87 days	Bilateral proximal limb weakness with possible muscle pain + / head drop, dyspnea, bulbar weakness, ophthalmoparesis or bilateral ptosis	Steroid myopathy; inherited and metabolic myopathies; other drugs-induced myopathies(statins)	-CK level -ENMG -Consider muscle biopsy	-High dose steroids -Monitor closely if respiratory muscle weakness	CK usually normalize in all patients within 44 days after therapy discontinuation

## Therapeutic strategies

As a rule, treatment consists of symptomatic management associated with steroids and/or either IVIg or plasmapheresis in severe cases. Mild (grade 1) neurologic symptoms do not require ICIs withdrawal, and the therapy can be continued under close observation. For grade 2 (or higher) *NAEs*, ICIs suspension is recommended in addition to steroids administration (ranging from prednisone 0.5–1 mg/kg to 1 g of methylprednisolone for 5 days) coupled with IVIg or plasma exchange as needed, depending on severity. In addition to corticosteroids, pyridostigmine titration from 30 mg three times a day to a maximum of 120 mg four times a day can be helpful in case of myasthenia gravis. Pregabalin, gabapentin, and duloxetine can be offered to relieve neuropathic pain [55]. Meningitis or encephalitis can be treated with empirical antiviral or antibiotic therapy if the drug-induced etiology is uncertain. There is no consensus on ICI-related MS relapses, but many studies report the classic 500 mg/1 g intravenous methylprednisolone for 3–5 days as a reasonable option [33].

Tocilizumab (anti IL-6R) has anecdotally been described as useful in blinatumomab and CAR-T cell-related encephalopathy (mostly in the setting of a diffuse CRS) [16, 30].

For a complete overview on diagnostic and therapeutic management, we recommend consulting *Management of Immune-Related Adverse Events in Patients Treated with Immune Checkpoint Inhibitor Therapy: American Society of Clinical Oncology Clinical Practice Guideline* published by Brahmer et al. [55]

Lastly, there are very few available data concerning the best therapeutic strategies in patients experiencing *NAEs* in whom immunotherapy cannot be suspended. Of utmost interest, natalizumab seems to decrease CNS inflammation without compromising the immune response against the tumor so it can prove to be a valuable option for these patients [56].

## Prognosis

The prognosis is extremely variable, depending on several parameters like *NAEs*’ severity (grading 1–5), the quality and the promptness of therapy administration, the patient’s age, and the prior performance status. Although the available literature reports no unequivocal data, Spain et al. suggest that about 33% of patients recover with sequelae [20].

### Encephalitis

Encephalitis generally improves spontaneously after treatment suspension but it is sometimes necessary to start one of the aforementioned therapies [57]. In a pharmacovigilance study by Johnson et al., the average post-encephalitis death time was 60.8 days, occurring mainly in the mixed encephalitis/aseptic meningitis cases (5 cases reported) [24]. As for blinatumomab, most cases resolved after treatment withdrawal [26].

### Aseptic meningitis

Most patients respond to corticosteroid therapy [23]. In most severe cases, the average time to death was 42 days in one study [24].

### Multiple sclerosis

In the series presented by Garcia and colleagues encompassing 14 patients, symptom resolution was reported in five patients with a median time of 8 weeks, whereas three patients experienced MS progression and two patients died. Data on the other patients were not available [34].

### Transverse myelitis

Several authors described transverse myelitis following treatment with ipilimumab. When administered after ipilimumab, corticosteroid therapy shortened the average recovery time to 2 weeks [40].

### Peripheral neuropathy

In the series by Dubey et al., corticosteroid use was associated with improvement in median modified Rankin Scale score and 70% of patients registered improvement on Inflammatory Neuropathy Cause and Treatment (INCAT) score. Rechallenge with the same ICI or switching to another ICI were both associated with a high risk of relapse [46].

### AIDP and CIDP

Drug suspension plus plasmapheresis or intravenous immunoglobulin (IVIg) treatment generally leads to symptom regression within weeks [40]. In patients with polyradiculoneuropathy or meningo-radiculo-neuritis with facial diplegia, symptoms improve rapidly (about 2 weeks) after high dose corticosteroids or IVIg administration [40, 56].

### Myasthenia gravis and myocarditis

The highest percentage of deaths was reported in patients developing myasthenic or myasthenia-like disease with concomitant heart (myocarditis) as well as muscles (myositis) involvement. In fact, as many as 62.5% of deaths (5/8) occurred in patients with both heart and muscles involvement, whereas fatal complications were rarer in patients with no myocarditis or myositis (16.2%), isolated myositis (20.7%), or isolated myocarditis (33%) [24]. Cardiac arrhythmias and acute myocardial infarction were the most common reported causes of death even in patients with no clinically obvious signs of myocarditis. These data suggest that several cases of myocarditis go underrecognized. We therefore recommend patients with ICI-related myasthenia gravis to undergo diagnostic evaluation for myocarditis and myositis using CK, troponin I level, and echocardiogram, as needed, even if asymptomatic [24].

### Myositis

Several studies report a mortality to be significantly higher in patients with ICI-related myositis rather than with idiopathic autoimmune myopathies (21.2%), while severe complications (defined as prolonged hospitalization, life-threatening event, or residual disability) occur in 49.4%. Most likely, though, these data need to be analyzed taking into account cancer-related events of other nature and several concurrent *irAEs* such as colitis, hepatitis, and, most notably, myocarditis [45]. In non-complicated cases, CK levels usually normalize in all patients after a median of 44 days of therapy discontinuation and recovery is generally good [54].

A practical overview on incidence, timing, clinical features, differential diagnosis, suggested diagnostic work-up, treatment, and prognosis is covered in Table 1
